# Photobiomodulation LED Devices for Home Use: Design, Function and Potential: A Pilot Study

**DOI:** 10.3390/dj13020076

**Published:** 2025-02-10

**Authors:** Mark Cronshaw, Steven Parker, Omar Hamadah, Josep Arnabat-Dominguez, Martin Grootveld

**Affiliations:** 1 Leicester School of Pharmacy, De Montfort University, Leicester LE1 9BH, UK; p2575563@alumni365.dmu.ac.uk (S.P.); mgrootveld@dmu.ac.uk (M.G.); 2 Department of Oral Medicine, Faculty of Dental Medicine, University of Damascus, Damascus P.O. Box 30621, Syria; omar.hamadah@damascusuniversity.edu.sy; 3 Faculty of Medicine and Health Sciences, University of Barcelona, 08907 Barcelona, Spain; joseparnabat@ub.edu

**Keywords:** photobiomodulation, LED device, home use, self applied, dental

## Abstract

**Background/Objectives**: Many commercial light-emitting diode (LED) devices are available for consumer home usage. The performance characteristics in respect to the dosimetry of many of the devices, currently on direct sale to the public, have not been subject to formal appraisal. In order to ‘bridge the gap’ between the evidence-based photobiomodulation therapy (PBMT) community and other interested parties, an evaluation is made of a selection of torch type hand-held LED PBMT products currently available for home use. **Methods**: Five randomly chosen intra-oral and hand-held LED PBMT devices were selected. The optical delivery parameters of the devices were measured, including the beam divergence angle, surface area exposure as well as the output power at the level of the LEDs. The surface and sub-surface temperature changes in porcine tissue samples were assessed under standardised conditions. The manufacturer’s patient instructions were correlated to the measured optical parameters. Calculations were made of irradiance and surface radiant exposure. Consumer satisfaction ratings and feedback data were collated, and a relevant statistical analysis conducted. **Results**: The results were heterogeneous with a wide range of applied wavelengths, output power and irradiance. Power output stability was variable, and, together with a wide beam divergence angle of 74°, the manufacturer’s directions for dosimetry were found to be inconsistent with an accurate dose delivery. **Conclusions**: The manufacturer’s proposed dosimetry fails to consider the relevance of the beam divergence angle and optical attenuation in view of the scatter and absorption. Appropriate instructions on how best to gain and optimise an acceptable clinical outcome were inconsistent with an evidence-based approach. Subject to validation by well-planned clinical trials, the concept of home PBMT may open interesting new therapeutic approaches.

## 1. Introduction

There are many conditions for which photobiomodulation therapy (PBMT) has been proposed to be of clinical benefit, including enhanced wound healing, as well as a useful management strategy to mitigate pain and inflammation [1,2,3,4]. However, there are logistical and financial challenges to the provision of such services. The practicalities associated with applying PBMT require access to equipment which may be expensive, particularly if additional units are required for a busy multi-surgery clinic. Also, many of the devices applied in the accepted PBMT evidence base are type IIIB/IV lasers, which have to be applied by trained certified clinicians within a treatment-controlled area, with wavelength-appropriate optical protection for all those in the treatment area. In order to satisfy the requirements as defined by National and International authorities, there is a panoply of laser safety regulations to fulfil, which includes the need to demonstrate compliance to the many aspects of training, maintenance, audit trails and safe utilisation [5]. Given these practical considerations, although the use of PBMT has been found to be a highly successful management tool for some clinical conditions, such as, for example, the prophylaxis of oral mucositis, the uptake on a worldwide basis has been slow to be adopted [6].

In consequence, efforts have been made to investigate the potential benefits of light-emitting diode (LED) devices as an alternative to lasers [7,8,9]. An LED as a source of optical emission offers a number of important differences to a laser. Indeed, a typical laser is characterised by its monochromatic property of single value wavelength, whereas an LED is typically a broader band-width emission wavelength source with a span of 30–50 nm. Additionally, lasers emit an intense coherent source with a stream of photons, in phase both spatially and temporally. In the absence of a corrective system of optics, the beam divergence angles of a laser most generally used for PBMT from our own measurements are typically around 30–35°. In consequence, a laser has a beam that is strong and concentrated, which can pose substantial optical hazards [10,11]. In contrast, LEDs are an incoherent, broader spectrum chaotic directionality source of a lower intensity and represent a correspondingly lowered risk of photothermal and photo-induced optical damage. LEDs represent a less costly option than a laser with a reduced regulatory requirement. Also, with appropriate instruction, it may prove possible for patients to be guided to self-treat in order to promote and enable prophylactic and pro-healing biological pathways, in addition to alleviating pain and inflammation. It is generally accepted that both LEDs and laser light sources can have considerable merit as photonic sources in PBMT. The integration of self-administered PBMT may prove to be a useful adjunct to many clinical processes.

There are, however, substantial differences between LEDs and lasers regarding potential for the delivery of photons to significant tissue depths. Indeed, with respect to optical physics, peak power is an important determinant of the delivery of photons in such a manner. By convention, the depth of optical penetration is described by 1/e, which represents ~<37% of the surface administered average power [12,13,14,15,16]. The optical transport of photons from a coherent narrow spectral range laser light source is a markedly different event to the more diffuse incoherent low intensity irradiation of an LED, and this is attributable to the inherent properties of laser light, as opposed to that of an LED. A laser source delivers energy, which is a very intense monochromatic and unidirectional beam of light. The possible added value of a laser source is an increased capacity to deliver photons to depth within a shorter period of time [17].

Given the many published studies supporting the benefits of PBM in cellular, animal and an increasing number of human clinical studies, there is considerable public interest to gain access to therapeutic devices which hold great promise [2,3,4].

Many PBMT LED devices are currently freely “available to buy” by consumers. Claims of positive clinical effects are made by retailers and manufacturers with respect to the amelioration of inflammation, muscle and joint pains, the treatment of herpetic and aphthous ulceration, angular cheilitis, oral candidiasis, and dentine sensitivity, together with the promotion of wound healing associated with gingival inflammation, oral wounds, and recovery from oral surgery.

For a medical device to be legally promoted for retail sale to the public, there are requirements within current legislation as determined by the appropriate national or regional regulator. In accordance with the USA Food and Drug Administration Modernization Act of 1997 (FDAMA) and the 21st Century Cures Act of 2016 (Cures Act), in order to permit the FDA to focus resources on higher risk items, and, in recognition of the lower risk and potential gain of phototherapy to public health, a category of product code ILY permits the listing of phototherapy type 2 medical devices as 510(K) exempt [18].

In Europe, however, there are complex regulatory pathways associated with the permitted access-to-sale to the public, of devices described as having medical benefits. In accordance with the stipulations of the EU medical devices directives, along with the evidence of good company and manufacturer governance, the evidence of independent safety tests in accordance with approved international standard agencies must be submitted. These evaluations include toxicity, allergy and sensitization tests, which are essentially the same as the ISO standards expected by the FDA for the 510k listing [19]. The key point of difference is that there is at present no sub-category of extended pre-market notifications in Europe similar to the FDA 510k exemption code ILY.

For a medical device sold to the public within the EU to make claims about clinical benefits, there is a statutory obligation to comply with the full legal requirements. Sanctions for non-compliance following an enforcement notice make provisions for this as a criminal offence, with associated penalties including fines and imprisonment.

“Red light” and near infra-red phototherapy devices can be currently purchased with ease from online internet suppliers, including mainstream retail sites such as Amazon, eBay and Taobao. The logistical challenges to the integration of PBMT as an element of directed clinical care may, in principle, be reduced by self-administered LEDs prescribed for home use. However, a number of key questions must be addressed as part of a professional duty of care prior to a clinician recommending an LED device to patients. Can the claims for clinical gain made by the manufacturers and distributors be acceptable as evidence-based? What instructions and support materials are provided to the user? What are the parameters recommended for use, and do these match the performance characteristics of the device? In order to explore these issues, this study was undertaken to appraise the claims made, the performance outcomes as reported by consumers, inherent and variable dosimetry parameters, the design in relation to proposed applications, and support materials for a sample of devices currently available for public purchase.

In the absence of the relevant supportive literature in dentistry for these hand-held devices, the objective of this paper is to attempt to bridge the gap between the evidence-based scientific community and other interested parties. The concept of home delivery PBMT devices may open new therapeutic possibilities. The secondary intention of this paper is to discuss within the context of the greater literature whether or not this type of device may have potential future value as a practitioner prescribed self-administered PBMT.

## 2. Materials and Methods

A survey of UK and USA internet was conducted of store sites (https://Amazon.co.uk (accessed on 2 June 2024) and https://Amazon.com (accessed on 2 June 2024)) offering oral PBMT devices. The filter terms “red light”, “phototherapy”, “photobiomodulation”, “oral” and “dental” were applied.

All devices were evaluated and enumerated, based on available data online for their stated purpose. A representative selection of five similar LED devices with consumer feedback following recent sales were included. Evidence was sought, where available, of compliance to regulatory requirements, including the display of a CE mark, the UK CA equivalent or FDA 510(K) clearance or exemption. The presence or absence of a claim of medical benefit was noted along with any disclaimers.

In the event that such information was missing, the distributor or manufacturer was contacted for further information. Consumer feedback as presented on the Amazon UK site is ranked on a rating scale low to high of 1–5*, and feedback comments from consumers were correlated with the star rating and assessed by two independent researchers (MC and SP) to identify common supportive or critical comments. Samples of 5 models of hand-held LED devices, all with current evidence of availability and sales, were selected and purchased online. The inherent and variable parameter characteristics of the devices were recorded and defined according to wavelength(s), operational modes (gated and continuous modes), manufacturer pre-set timers, along with the provision of instructions and safety glasses. The content of the instructions was evaluated on a 6-point scale of 0–5, where zero represented a total absence of instruction, 1–2 basic generic operational instructions, 3–4 the presence of advice on operation for differing conditions, and 5 as fully comprehensive advice with links to other educational and user support services. Key points of advice to the consumer on the choice of wavelengths, emission modes, application times, the frequency of usage, and distance to the target tissue area were recorded.

The output power of the devices was measured five times each, using a power meter (Thor P160 ThorLabs, Newton, NJ, USA) of all available wavelengths and combinations of wavelengths. A record of the output power at intervals of 60–300 s whilst following the manufacturer-determined maximum exposure time of each device was also made. Devices with gated modes and added removable applicators such as glass LED guides were further assessed for power output with the attachment in place.

**Figure 1:**
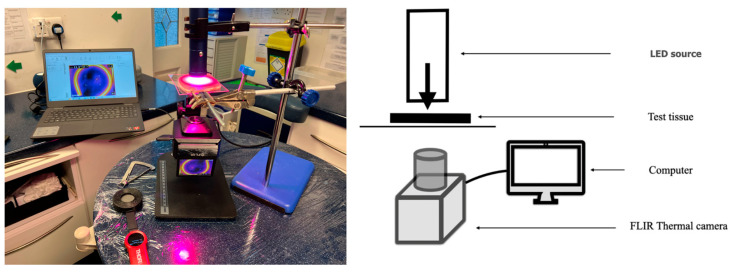
Test set-up. LED source irradiates test tissues. Thermal measurements (FLIR ETS-320 camera) recorded and analysed (FLIR Thermal Studio Pro, Teledyne FLIR, Wilsonville, OR, USA). Transillumination: thermal measurements are taken of seven sets of samples. Surface thermal measurements are taken immediately before and after radiant exposure at the manufacturer’s preset cut off times of three minutes (Kinreen, device 5) and five minutes (Homesta, device 4).

Thermal measurements of 7 sets of readings were taken using a FLIR ETS-320 camera (FLIR Thermal Studio Pro, Teledyne FLIR, Wilsonville, OR, USA) of the surface of samples of lean porcine muscle tissue localised in near contact with the sample tissues, as well as during the transillumination of standardised 5 mm thick lean porcine tissue sourced from a local retail outlet. The thickness of each tissue sample was verified on the basis of three measurements using an Iwanson calliper gauge under 4× magnification to a tolerance of +/−0.5 mm. Details of the test jig set-up are illustrated below (Figure 1) and are the same as our earlier published study [20].

Measurements of the beam divergence angle were made, and calculations of the irradiance and fluence were recorded. Furthermore, the measured fluence was cross-referenced to the manufacturer’s dosimetry advice to consumers. The level of regulatory compliance was recorded, and, where necessary, the manufacturers or their distributors were contacted to determine the same. Where provided, the safety glasses supplied with the devices were assessed to measure the level of beam attenuation, in addition to recording any indications of this on the eyewear frames.

Statistical analyses were conducted of the consumer satisfaction ratings, power outputs and thermal measurements of each of the devices. The non-parametric Kruskal–Wallace test was applied to explore the product and study dependence of individual numbers of participant Likert rating scale values using the *XLSTAT2024* software option (Addinsoft, Paris, France). A corresponding parametric one-way analysis of variance model was also employed for the analysis of these data. Additionally, two separate χ^2^ contingency table analyses were performed using this software option, the first featuring results acquired from all separate reported studies, the second with only LED product-dependent (summated) Likert score values from all studies available on these products. Wilks’ G^2^, Fisher’s exact test and relevant permutation statistics were also computed. For the analysis of power outputs and thermal measurements, both one- and two-way ANOVA models, and matched two sample comparisons using paired sample *t*-tests, were also performed using *XLSTAT2024* software.

## 3. Results

**Figure 2:**
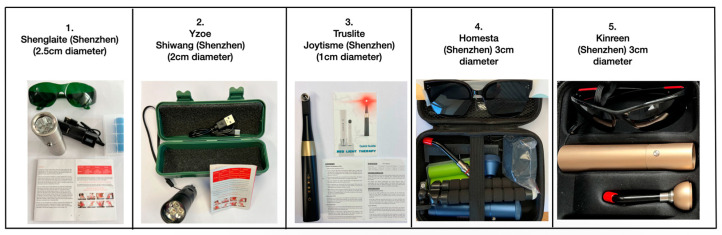
Names and images of devices include 1–5 plus accessories (protective eyewear and glass light guides).

Our evaluation of the basic characterisation of the various devices is shown below (Figure 2, Table 1 and Table 2).

Primarily, statistical analysis of the raw Likert scale dataset (model 1) with the non-parametric Kruskall–Wallis test revealed that there were very highly significant differences between each product’s median values (*p* < 10^−6^), and these were in the rating order Homesta (4) > Shenglaite (1) > Kinreen (5) > Yzoe (2) > Truslite (3). A corresponding parametric ANOVA analysis of these raw data also revealed a very high level of significant values between the least squares mean (LSM) values for each product, and this approach yielded the same ranking order. Indeed, mean ± 95% confidence interval (CI) rating scales for these products were found to be 4.31 ± 0.22, 4.18 ± 0.24, 4.04 ± 0.13, 3.75 ± 0.14 and 3.55 ± 0.15 for the Homesta, Shenglaite, Kinreen, Yzoe and Truslite devices, respectively; i.e., there were significant differences between statistically equivalent Homesta, Shenglaite and Kinreen, and the Yzoe and Truslite products only, but not between the latter two.

Subsequently, we analysed the dataset using two sets of χ^2^ contingency tables to monitor associations between each of the products investigated and consumer Likert score value responses, the first approach including all consumer review studies reported in this manuscript, the second, only the five products and their specifications; in this case, enumeration data form studies focused on the same product were summed and combined for this model.

**Figure 3:**
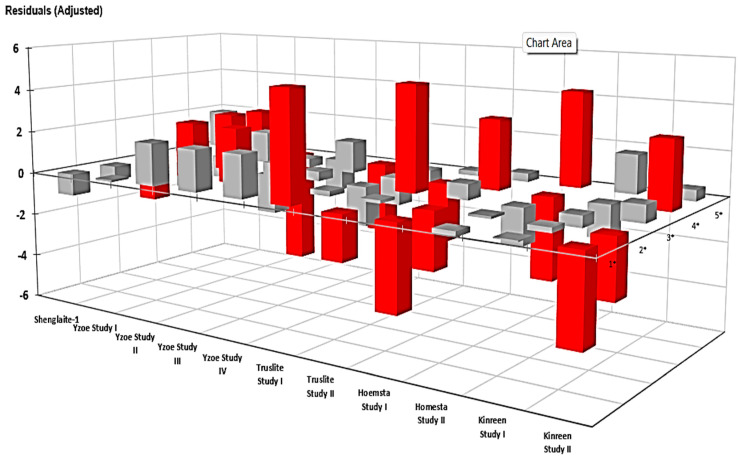
Plot of adjusted χ^2^ contingency table residuals (differences between observed and expected values) for the Likert scale score values acquired for each reported study conducted. These results clearly confirm the association between rows (product studies) and columns (Likert score values). Red and grey colour codes represent statistically significant and non-significant adjusted cellular residuals, i.e., raw differences between the observed and expected contingency table counts divided by their corresponding standard error estimates.

**Figure 4:**
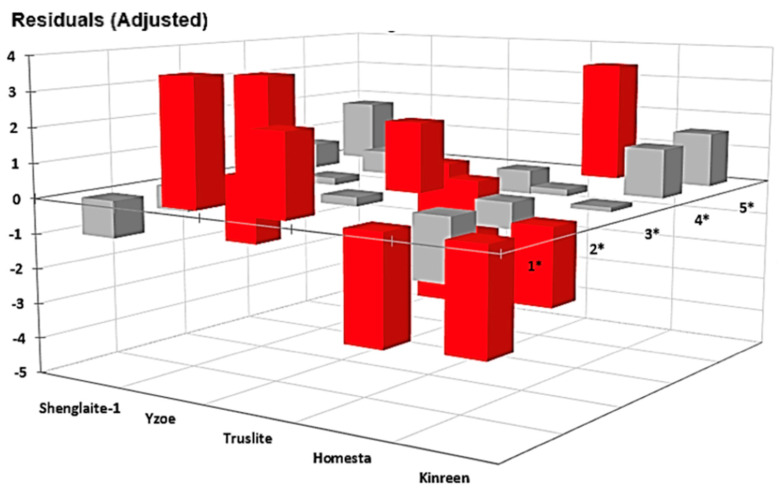
The plot of adjusted χ^2^ contingency table residuals (differences between observed and expected values) for the Likert scale score values acquired for each product evaluated (summated values for four of these). These results clearly confirm the association between rows (LED products) and columns (Likert score values). Red and grey colour codes represent statistically significant and non-significant adjusted cellular residuals, i.e., raw differences between the observed and expected contingency table counts divided by their corresponding standard error estimates.

Significant (*p* < 10^−6^) χ^2^ contributions for the first analysis showed that adjusted residuals, i.e., those remaining following subtraction of the observed data from that expected, not only differed between the series of LED products tested, but also ‘between-studies-within-products’ for all products had more than a single report on the efficacies of their devices in the scientific literature (Figure 3 and Table 3). Both Wilks’ G^2^ test along with a test of independence between the contingency table rows and the columns using the Monte Carlo method (with 5000 simulations) were conducted, and both these tests were found to be extremely significant (also *p* < 10^−6^). A larger number of statistically significant deviations were observed at the lower Likert rating scale values. As expected, when such studies were combined in assessments for each product only (*p* < 10^−6^), the differences between them remained (Figure 4 and Table 4), and, again, more of them were associated with the lower Likert scale ratings.

**Figure 5:**
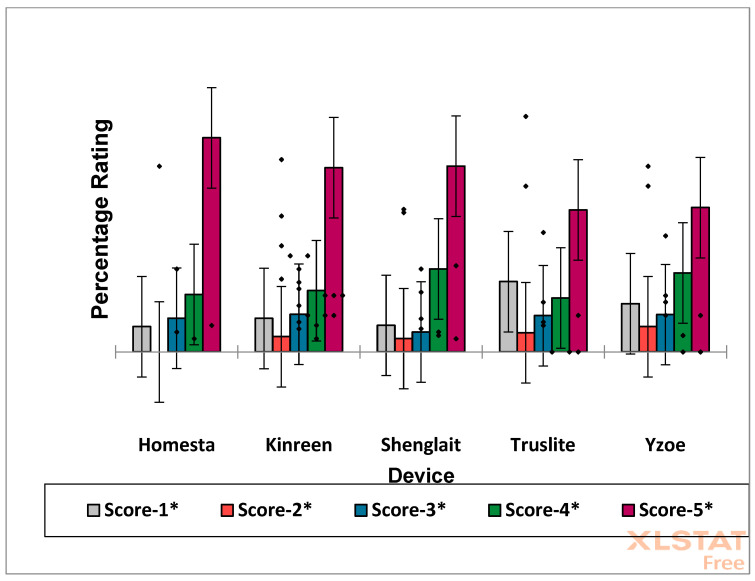
Bar diagram plots of mean ± 95% confidence intervals for percentages of consumer score ratings for each of the five different products evaluated.

Further analysis revealed that, for all the consumer response data explored, the mean 5* rating was highly significantly more greatly selected than all the others; the mean 4* rating was highly significantly greater than the mean 3* and 2* scores, but not the 1* one; the 1* mean rating was more popular than the 2* but not the 3* one; and the 3* mean score was rated significantly more highly than the corresponding 2* value (Figure 5).

With regard to the manuals supplied by the manufacturers, the contents refer primarily to pain mitigation with a range of recommended treatment times daily, varying between 1 and 20 min for each dose. Distance of the device to the tissue target surface varied, from images showing the application in close proximity to the affected area to advisory materials in the manuals to apply the beam: “at a distance of 1–3” inches away” (~2.6–6 cm) from the skin surface. Some images show the use of a glass light guide introduced intra-orally as well as intra-nasally.

Dependent on model, the operator variable options included a choice of wavelengths and multiple simultaneous ones. Also, some models offered a gated (“flashing”) mode with a 50:50 10 Hz on/off emission cycle (Table 4). The stated benefit in the manuals of the gated modes is “to reduce physiological stress cells already under oxidative stress”; also, another benefit is that this mode “is better for cell stimulation and recovery”. Another manual proposed that gated modes enhanced optical penetration into deeper tissues.

Application times varied according to the device and clinical intent, which were dependent on the severity of pain relief sought. The frequency of application varied in range from 1 to 6 times daily with the advice not to use the light for more than 5 min at a time and to allow 1 min for the device to cool down before any further applications.

A qualitative assessment of the consumer instruction manuals and links to other support materials scored from 1 to 5 (basic generic advice to limited specific advice by condition). Very little detailed guidance is offered to the consumer other than to propose more frequent and longer duration therapies as an aid to the management of ascending levels of pain (Table 1).

**Figure 6:**
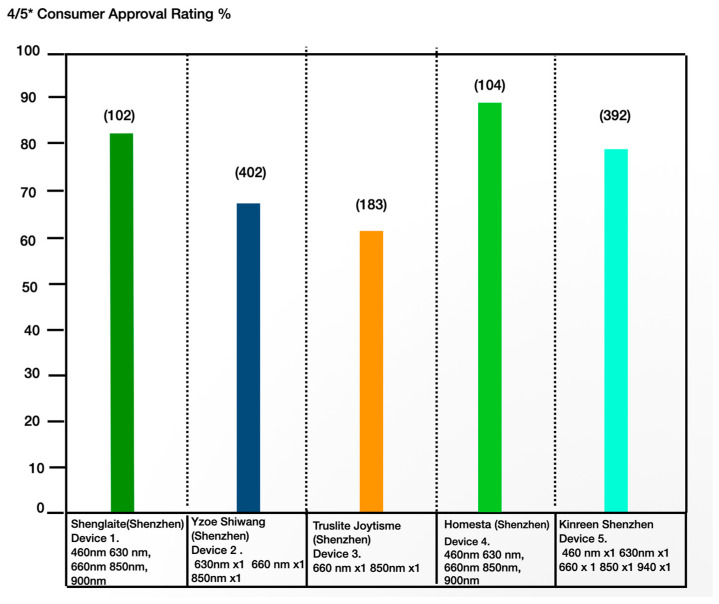
Consumer approval ratings 4–5* for the five devices (numbers of participants in brackets).

The overall level of consumer satisfaction was high as indicated (Figure 6), along with many feedback comments attesting to pain mitigation, good wound resolution, in addition to the effective treatment of herpetic lesions.

**Figure 7:**
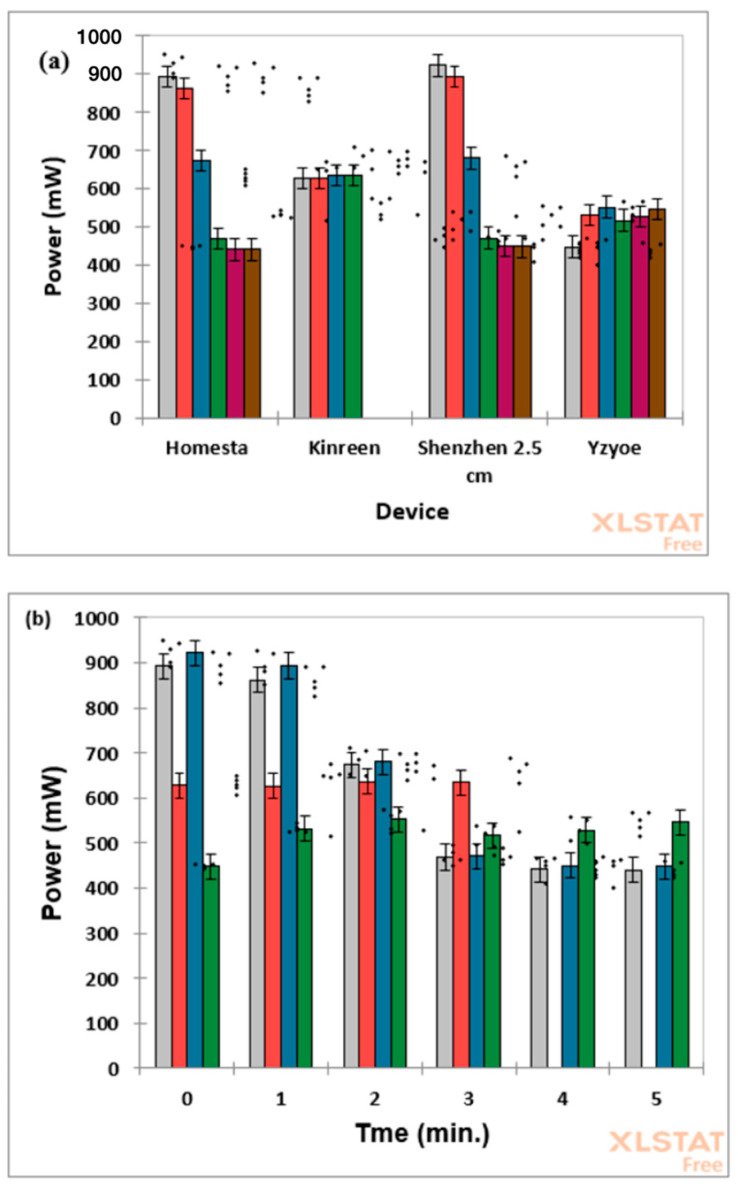
Mean ± 95% confidence interval power loss value bar diagrams for the LED devices evaluated. Plots for (**a**) devices and (**b**) sampling time points are plotted. Power measured at time intervals of 1.00 min and was based on 5 sets of readings for each device. Colour codings for (**a**) grey, 0.00 min; pink, 1.00 min; blue, 2.00 min; green, 3.0 min; purple, 4.0 min and brown 5.0 min. Colour codings for (**b**) grey, Homesta; pink, Kinreen; blue, Shenglaite; and green, Yzoe.

Measurements of power output and a calculation of the irradiance for each device are itemised in Table 1. It was found, however, that the power output of the devices were not constant over time as illustrated (Figure 7).

As observed, the power reductions associated with the Shenglaite (device 1) and Homesta (device 4) devices were approximately 50% of the initial power output at 3.00 min. In contrast, the Kinreen Shenzhen (device 5) maintained a stable output up to the manufacturer-set 3.00 min limit of exposure.

Statistical analysis using a two-factor fixed effect ANOVA design (with an associated interaction term) demonstrated that differences exist between the mean values of both the ‘between-product’ and ‘between-time-point’ main factors (*p* = 4.11 × 10^−8^ and 2.33 × 10^−50^, respectively); additionally, the two-factor interaction effect was also very highly significant (*p* = 7.01 × 10^−45^). The significant interaction term in this model is readily explicable to differential time dependencies of the power outputs from each of the products tested. Indeed, for the Shenglaite (device 1) and Homesta (device 4) products, this value appears to decrease in a sigmoidal (S-shaped) manner, whereas that for the Kinreen (device 5) remains invariant with time points for up to 3.0 min. Moreover, the power versus time-point relationship for the Yzoe (device 2) product increases within the first minute and then remains approximately constant from the 2.0–5.0 min time points.

For the Truslite (device 3), a paired sample *t*-test performed on the limited data available showed that there was a statistically significant increase in power after a 1.00 min duration (*p* = 0.0054).

The thermal measures made revealed a rise in temperature in both the surface and sub-surface porcine tissues. Our results demonstrate a surface temperature rise on LED activation in the range of 5.7–7.8 °C and a temperature rise at 5 mm depth in the range of 4.1–6.2 °C at the 180 s time point, with the selected Kinreen device emitting five simultaneous wavelengths at an irradiance of 106 mW/cm^2^. This difference is very highly statistically significant (*p* = 3.25 × 10^−4^, paired sample *t*-test). Following a 5 min emission period with all five wavelengths simultaneously, the Homesta torch device metallic shell increased from an ambient surface temperature of 20 °C to within the 32–44 °C range with the temperature ascending to 44 °C on repeated immediate reactivation.

**Figure 8:**
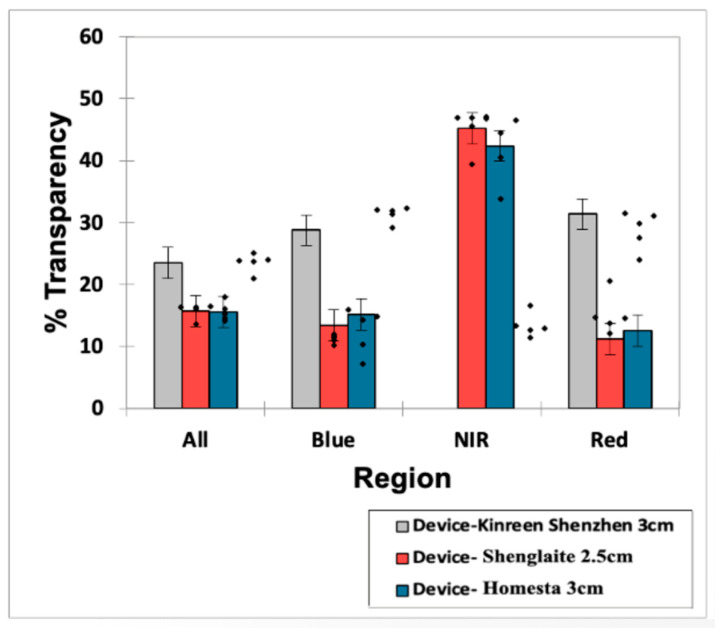
Effect of optical safety glasses. Bar diagram of mean ± 95% CIs for the percentage beam transparencies of three devices tested (mean values are those for 5 sets of readings).

Safety glasses were provided for three of the five devices There were no indications of any specific markings for the level of optical protection provided. The specification of the build of the glasses did not include side protection panels, and, in essence, in build and quality, they are equivalent to basic sunglasses. Overall, the measured level of optical attenuation by wavelength and device ranged from 55 to 88%, which of course corresponds to 12 to 45% transparency, as shown in Figure 8. Statistical analysis conducted using a two-factor model with an interaction term found that the ‘between-products’ and ‘between wavelength regions’ mean squares values were both extremely significant (*p* = 6.75 × 10^−19^ and 6.41 × 10^−29^, respectively), although the associated first-order interaction effect was also highly significant (*p* = 1.03 × 10^−4^), with the latter reflecting non-additivity in the model, i.e., differential responses of the three products evaluated at each of the wavelength region studies (Figure 8).

## 4. Discussion

The outcome of this study indicates a high level of consumer satisfaction with the home delivery LED PBMT devices included in this report. Many consumers purchase a torch type device primarily for non-dental orthopaedic applications, although many report using it for intra-oral and peri-oral therapies, including herpetic lesions and oral ulcers, as well as the treatment of head and neck myalgia (Table 1).

The current peer-reviewed evidence base does not specifically extend to clinical trials of device benefits, nor otherwise of the use of hand-held torch type LED PBMT devices in dentistry. Prior interest recorded in the literature for patient self-administered PBMT LED devices in dentistry in part relates to orthodontics for pain control and orthodontic tooth movement acceleration [21,22,23]. Also, there are reports related to the management of complications of oncology, chemotherapy and radiotherapy such as oral mucositis and xerostomia [24,25]. However, none related to torch type devices were identified. Furthermore, the unit costs of the devices described in these studies are considerably higher (in the range of GBP 1200–25,000), and none were available for direct sale to the public. By contrast, the torch type devices are much less expensive to purchase. In a 2019 review, Gavish and Houreld comment that the majority of home use LED devices described in the literature at that time referred predominantly to facial aesthetics and some dermatological conditions [9]. At present, other than in orthodontics, there is at present very little in the dental literature on home use PBMT. Given the relatively low cost and accessibility of the devices plus the evident consumer enthusiasm, this type of appliance clearly warrants further professional objective analysis and qualification.

Our results showed that there was a range of output powers and irradiance available with many operator options. Depending on each device as itemised, these included a choice of wavelengths and variable parameters, including application time, optical spot size at the target, and gated modes. Two of the devices have an optional glass optic guide to assist dose delivery.

However, the distributor’s and manufacturer’s support materials are far below the level of information required to enable the user to achieve defined clinical outcomes. Indeed, although the parameters of the various devices varied considerably, three of the five devices tested here had an instruction manual which was essentially the same document. The exceptions to this were the Truslite Joytisme device, which has evidently been designed primarily to treat herpetic eruptions and a limited range of oral ulcer and localised dermatological uses, e.g., acne. Also, the multi-wavelength Homesta 3 cm device has some links to helpful videos and a few recommendations on the treatment of arthritis, tendonitis, acne, herpetic lesions and oral ulcers.

The information provided, however, is very basic. This deficit is a frequent issue referred to by frustrated consumers. Although there are positive reports of pain relief and more rapid wound resolution in some conditions, such as in the outcome reports on labial herpes simplex, the marketing of a universal solution and approach to pain management within the limits of the advice given is a statement of hope, as opposed to a reliable and predictable evidence-based outcome approach.

**Figure 9:**
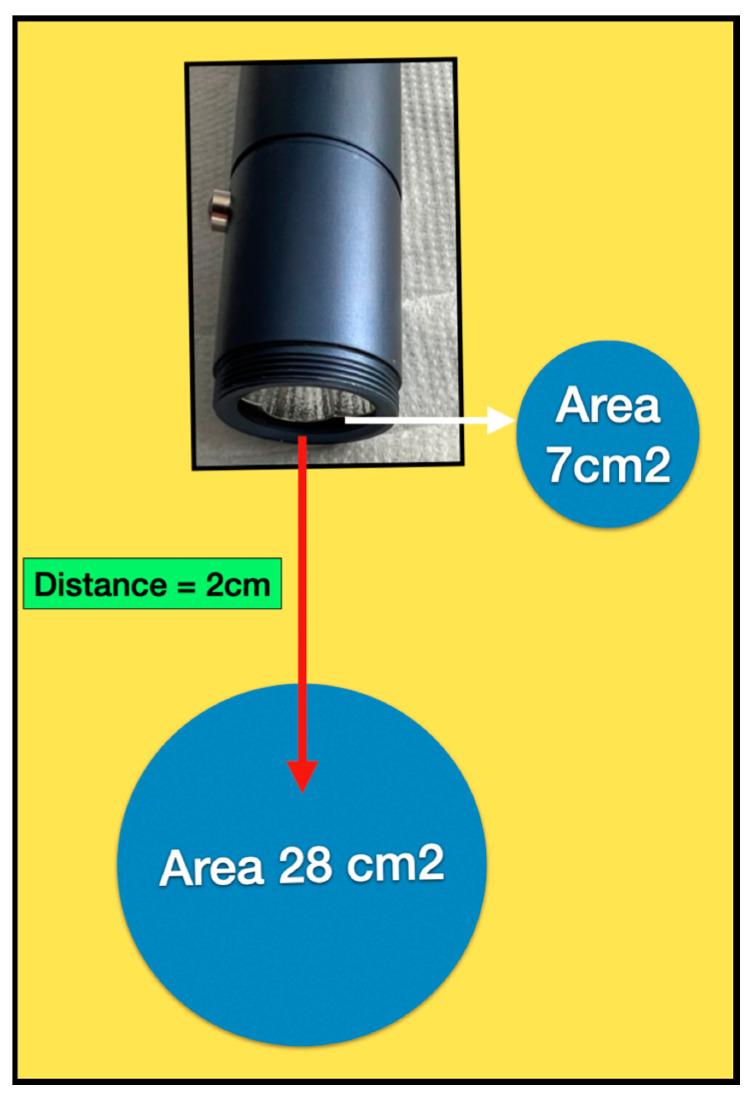
For the purposes of illustration, if an LED source with a diameter of 3 cm has a beam divergence angle of 70°, then, at a distance of 2 cm away, the area of exposure is around 4-fold the spot surface area of the device (28 cm^2^).

**Optical Parameters:** Our analysis of the parameters recommended to treat clinical conditions, and their relation to the inherent and user variable parameters, indicates a lack of appreciation by the manufacturers of dosimetry with respect to optical transport pathways. This was apparent in the advice to users to apply the light from a distance ranging from near contact to 7.5 cm away. Indeed, the beam divergence angle of four of the five devices was around 74°. In consequence, the area of exposure of the beam varies considerably in relation to the proximity to the target area. In near contact, a 3 cm diameter device has a surface area beam exposure of approximately 7 cm^2^, whereas, at a distance of 2 cm, the area of the beam is four times higher at 28 cm^2^. At a distance of 7.5 cm away, the beam area is 154 cm^2^. Hence, the irradiance varies dramatically with distance from the target surface. This has a considerable effect on the time-dependent radiant exposure (Figure 9).

**Figure 10:**
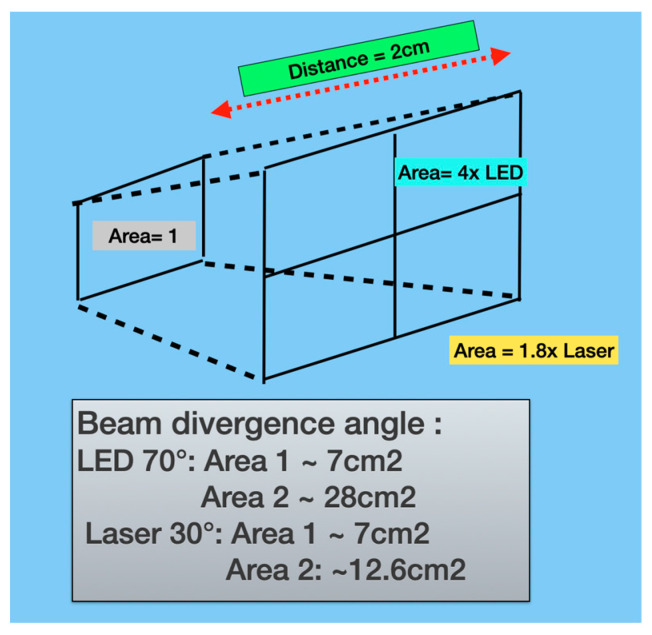
A laser with a 3 cm diameter beam at a source and beam divergence angle of 30°, applied at a distance of 2 cm, has a spot size of <2-fold the source (12.6 cm^2^). In consequence, the laser has an average irradiance of ~50% of the source. In contrast, the average irradiance (W/cm^2^) of the LED at the target is <25% of the emission source.

Inherently, a laser source has a Gaussian power distribution across the beam, with a beam divergence from the source of typically around 30° (15° each half-side). By contrast, an LED has a much wider beam divergence angle typically of around 70° (35° each half-side). In consequence, the area of exposure with an LED at a distance from the source is considerably greater than with a laser source, with far less energy concentrated in the mid-third of the beam (Figure 10).

**Figure 11:**
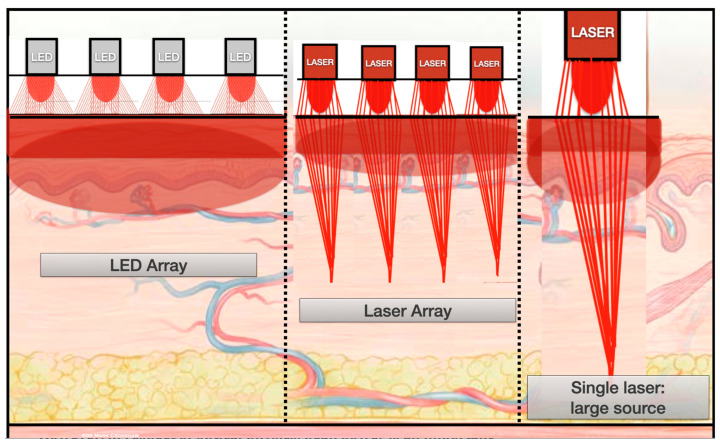
The wide beam divergence of an LED source increases the area of surface exposure and reduces the peak power concentration in the mid-third of the beam, unlike a laser source. However, a laser has a narrow spectral range with a coherent waveform, and the light waves move forward together both in time and space.

Many LED PBMT devices employ multiple small point sources as an array, either arranged in the torch type devices as a cluster or, alternatively, spaced in a flat panel or a flexible fabric material. This may conceivably permit a more even surface dosimetry with a saturation of photonic exposure to a larger superficial area by LED arrays, in contrast to a multiple small point laser array or a single larger laser applicator point (Figure 11). However, an LED is regarded as a ‘chaotic’ light source with multiple non-aligned photon streams of a broader spectral range. In contrast, a laser has a tight beam that is strong, concentrated and better able to penetrate tissues to a greater depth. Furthermore, the opto-physical effects of the simultaneous impact of a laser photonic source at the molecular level is inherently more profound. Whether or not this may result in differing clinical observable effects is a continued area of discussion within the PBM research community [7,8,17,26,27].

**Regulatory Compliance:** There are very many different types of PBMT devices currently on sale direct to the public. These include LED embedded flat panel devices, LED-lined wrap-around blankets and caps, LED-lined face masks for facial aesthetics, and intra-nasal devices, amongst many other designs. Few if any have attained a CE medical device listing, although nearly all have the basic legally required electrical safety certification. In contravention of the EU medical devices directive requiring CE accreditation, all devices we appraised for this study stated medical benefits in the instructions supplied with the devices, as well as on their respective internet sites. Presumably in recognition of the EU legal requirement, our results showed that four of the five devices had a disclaimer to health gain. However, this could be found in the discrete small print details of the distributors’ web pages, whereas the proposed health benefits were prominently displayed.

In the USA and other global regions such as the EU, any adverse issues reported could trigger a compliance requirement, with the possibility of devices being recalled, and a fuller investigation instigated. In the absence of evidence of compliance to the required safeguards, and without a peer-reviewed supporting clinical trial, to prescribe one of these devices clearly poses a medico-legal risk to a practitioner.

**Optical Delivery Considerations:** Given the mismatch between declared purpose and performance with the devices currently available to consumers, is there a future for practitioner-prescribed patient self-administered PBMT? In our opinion, the answer to this question requires some careful qualification and consideration.

In a study investigating a trans-buccal approach to the treatment of oral mucositis using an 850 nm LED multiple point array, the direct measured power delivery of the surface applied power at a depth of 6–12 mm ranged from 0.6 to 0.2%. The validity of this outcome was further confirmed by a Monte Carlo model which agreed with the measured outcome to within 12%. Contrastingly, laser tissue penetration studies show energy delivery to an equivalent depth of around 1 cm of 3–10%, a parameter which is dependent on surface optical spot size, emission mode, wavelength and tissue consistency [28,29].

In accordance with the laws of photobiology, the optical transport of light to depth is regarded as a prime requisite to activate the photon transduction pathways associated with PBM. Since some pathologies are deeply seated within the tissues, the logistics of the dose delivery of photons to depth within a reasonable time frame represents a considerable challenge, which with a clinic-based system may be best accomplished by a laser. However, in accordance with the third law of photobiology known as the Bunsen–Roscoe law of reciprocity, it is acknowledged that a positive PBM effect can be achieved by protracted exposure to a lower intensity source. Shen et al., for example, found a positive bio-stimulatory effect on fibroblast collagen production in a tissue culture study on cells irradiated with a 634 nm LED luminous fabric source of 0.1424 mW/cm^2^ for 5.9 h [30,31].

There are many PBM studies based on 2D-planar mono-culture cell models. Furthermore, there are very many small animal studies which have proven useful to elucidate some of the pathways associated with PBM. However, translating the same to human subjects has represented an interesting challenge posed by the conceptual requirement to deliver photons to depth. Human studies employing LEDs as a surface-applied source have claimed positive results in treating some deep CNS pathologies; this has been the source of interest in the literature, since direct measurements of LED photonic penetration fall far short of the evident requirement [17,26]. Are the positive outcomes recorded in fact a placebo response, or are there other mechanisms involved?

**Possible Modes of Action:** There is a paradox of how LEDs can have a beneficial PBM response to sub-surface targets, in view of having limited tissue penetration of the applied photons. Indeed, it has been proposed that there may be an overlay between the recognised effects of infra-red heat therapy and a synergistic PBM effect on more superficial tissues [32].

At the molecular level, the absorption of photons has been proposed to have a highly localised photothermal effect on nano-structured water clusters, which may produce changes in fluid viscosity, as well as on the conformation of important protein structures such as enzymes and membrane-bound ion gates [33,34]. At higher temperatures, within the tolerance of the tissues to withstand thermal stress, this can induce an increase in cellular resilience, in addition to other hormetic physiological responses, which may be a contributory factor to analgesia.

It should also be noted that there are light-sensitive membrane bound ion gates known as opsins and pleiotropic responders to external stimuli known as transient receptor proteins (TRPs) [35,36]. The activation of TRPs include the opening of calcium and sodium ion channels into the cytoplasm and mitochondria, which can result in a wide range of important physiological effects, including contrary effects on axonal metabolism. Calcium ions represent key operands in stimulating mitochondrial metabolism, which may result in hyperalgesia. Notwithstanding, elevated cytoplasmic calcium ions can activate mitochondrial transport pores, resulting in a diminution of mitochondrial metabolism. Indeed, TRPs are recognised as relatively non-selective ion channels which may reduce the sodium–potassium ion gradient across the axonal membrane [37]. This, in turn, may impede the onward transmission of a wave of depolarisation along the length of the axon. The mechanisms of photon-induced analgesia are a matter of continued investigation [38,39,40,41].

In an earlier in vitro study, we found evidence of a photothermal response to a depth of 2–3 cm into test porcine tissues on laser irradiation [20]. Our results in this study demonstrate a surface temperature rise on LED activation in the range of 5.7–7.8 °C and a temperature rise at 5 mm depth in the range of 4.1–6.2 °C at the 180 s time point, with the selected device emitting five simultaneous wavelengths at an irradiance of 106 mW/cm^2^. By the non-radiative transfer of heat, this can be reasonably anticipated to have some physiological effects at depth into the tissues in vivo.

An elevation of tissue temperature up to 2 °C stimulates an increase in cellular metabolism, as well as vasodilatation, which increases the bioavailability of oxygenated blood [42,43]. Above a 2 °C increase in temperature, a hormetic response is activated via a system of heat stress proteins (HSPs) [44,45]. Furthermore, as cytoplasmic ROS levels rise, there is release and the increased gene transcription of a protective protein known as ATF-4, which reduces cellular metabolism and confers some additional protection against thermal stress-induced protein and fatty acid chain damage. Also, visible-to-near infra-red wavelengths of light are strongly absorbed by chromophoric transition metal ions such as those of iron and copper for example, which are at the core of important enzyme clusters such as cytochromes, cryptochromes, flavins and porphyrins. Even very low exposure to an incoming photonic source may have significant effects on electron transport, ionisation status, and difficult to measure highly localised thermal rises. However, there is agreement that PBM by definition is not primarily a photothermal event. Any temperature rise associated as a collateral effect of therapeutic PBM photon transduction should be kept below a sustained threshold level of 44 °C. Key anti-oxidant enzymes catalase and glutathione reductase are inactivated at temperatures above 43 °C, and, at even higher temperatures, there is an ascending risk of the permanent deformation of structural proteins and fatty acid chains [46].

A photothermal tissue response may be beneficial in temporarily relieving some of the symptoms attached to some inflammatory disorders when applied at lower levels. However, this is not the equivalent of triggering more enduring positively directed cellular and tissue response pathways associated with PBMT-promoted enhanced quality healing, repair and resolution. Nevertheless, it is recognised that there is a difference between a thermal source and PBM. Although both may be associated with an overt thermal rise, the physiological effects differ [32].This difference may be manifest as long duration photon-induced analgesia, as well as characterised changes in gene transcription pathways [37,38,39,40,41]. The temporary thermally induced palliation of distressing symptoms observed may be justifiably viewed as a positive outcome. However, with LED sources, the enduring value of the positive responses reported by consumers can only be viewed as indicative of the need to conduct properly designed clinical trials.

It is axiomatic that interlinked multiple overlying systems recognised as being operands in PBMT exist within a 3-dimensional highly integrated biological entity. A distant stimulus can trigger a local, regional and systemic response via autocrine, paracrine and endocrine signalling. Moreover, the central and peripheral nervous system can have a profound effect on cellular processes via the nociceptive A-δ and C fibres, sensory mechanoreceptors, along with the autonomic parasympathetic and the sympathetic nervous system. Since multiple operands may be affected, which in consequence effects overt changes in cellular physiology, there may be an outcome best described as “of entrainment” [47].

Such entrainment is recognised in the scientific literature as a phenomenon in which two or more oscillators interact with each other [47,48,49]. All electromagnetic sources have the property of variation in amplitude at a fixed periodicity according to wavelength. Oscillating systems can interact with each other in a number of manners. They can enhance or negate each other’s effects (constructive and destructive interference, respectively). Also, they can synergise with each other to achieve amplitudes greater than the sum of the two systems (resonance). When two or more oscillating systems interact, one or all can experience an alteration in frequency to become phase-locked, i.e., the phase difference between the oscillating systems remains constant in time and is robust to perturbations.

An example of photo-induced entrainment is the circadian clock which, in phase with the daytime active and night time sleep cycle, sets the time domain sequence of the sympathetic nervous system over the para-sympathetic nervous system [50]. Bright light stimulates specialised retinal receptors, and, via the supra-chiasmatic nucleus, this results in the inhibition of melatonin production within the pineal gland. Bright light can also have interesting effects on epidermal melanocytes when photo-stimulated melanocytes generate and release neurotransmitters, including serotonin, noradrenaline, and dopamine, in addition to adrenocorticotrophic, growth and luteinizing hormones [51]. Furthermore, blue wavelengths of light can generate nitric oxide from epidermal nitrosyl compounds resulting in vasodilatation [52]. Pathways of optical transmission may include vellus, blonde and grey hair, which have been proposed to potentially act as a photo-optic guide carrying light into the dermis [53,54,55]. Similar effects have been identified in dental tissues, since both enamel prisms and dentinal tubules can transmit surface-applied light to the pulp tissues [56]. Also, the myelin sheath surrounding the axonal fibre acts as a light conduit transporting biophotons to the axonal cell body [57,58,59,60]. The myelinated sensory and motor innervation of the epidermis and dermis is high in some parts of the body, for example, the hands, feet and peri-oral region, and there remains the possibility that the myelin sheath may facilitate the optical transmission of an external applied source. This concept may be worthy of investigation, since it may offer insight into factors associated with the positive benefits discovered in some CNS studies, for example, [61,62], as well, perhaps, as in laser acupuncture [63].

**Considerations of Reliability:** LEDs are recognised generally as being only around 20–30% efficient, and the power losses are manifested as heat. In this respect, the finding that the power output of two of the devices tested dropped by over 50% over the period of exposure is significant. In our opinion, it is noteworthy that the devices with the least stable irradiance had the highest overall power output. Indeed, it is recognised that the efficiency of LEDs is reduced by higher temperatures. Moreover, this may predispose to failure of the device LED chip, notably because solder junctions fail [64,65,66].

Also, in respect to consumer feedback, product reliability and function, it was of note that the negative feedback comments mentioned the device becoming hot to touch. Our measurements recorded a temperature range for the torch case of 32–44°C after a single 5 min cycle using the Homesta unit (device 4). In the instructions to patients, the manufacturers recommend allowing five minutes before a further cycle of emission. However, the torch case acts a heat sink for the LEDs, and, after the recommended five minutes delay, the device rapidly re-heated to 44 °C on reactivation.

**Optical Safety:** With regard to optical safety, the use of any visible spectrum bright light source may cause temporary discomfort; however, this is not regarded as a significant long-term risk with LED sources at wavelengths >550 nm. The blue wavelengths are recognised as a potential hazard in view of photo-retinopathy which is ascribable to the induction of intracellular ROS. Nevertheless, bright light sources produce a natural aversion response by the eye in most cases. This response limits the duration of exposure to a fraction of a second (typically less than 0.25 s). Beyond the visible spectrum at wavelengths of >780 nm, i.e., the near infra-red wavelengths of LEDs, at the irradiance measured in this study of 41–43 mW/cm^2^, can be regarded as well within safe limits. Direct exposure to a blue wavelength source is not advised, and the safety limit has been proposed as 30 mW cm^2^ for a 1000 s exposure to 441 nm blue laser light [66,67]. The blue 460 nm wavelength torch devices included in this study emit at an irradiance of 34–41 mW/cm^2^. Staring directly into the beam at close range to the eye could pose an optical challenge. However, because of the high beam divergence angle and the natural aversion blink reflex to a bright light source, this risk can only be regarded as small. However, some segments of the population, e.g., newborns, young children and the elderly may be more susceptible to some adverse biological effects from blue-rich LEDs. Infra-red and high brightness pure blue LEDs must be used with additional caution, since exposure to the glare will stimulate a reduced aversion or nil autonomic response to the optic hazard. LEDs, whether visible or IR applicators, are more akin to lamps in terms of the spectral bandwidth emission profile and radiance, and, since they are not inherently intense coherent sources (lasers), they are safe under reasonably foreseeable usage conditions for acute exposure.

In view of this, some form of optical protection may reasonably be viewed as an appropriate measure for babies, young children and older adults. The eyewear supplied by the manufacturers is of a poor build quality, however, and the level of optical attenuation is adequate but not high and is comparable to inexpensive sunglasses. Indeed, they are not compliant with the IEC specifications required for designated optical safety glasses, although they do confer some basic protection. However, pending any future change in legislation with regard to LEDs, they do at least represent some form of recognition by some of the manufacturers for the desirability to have available protective eyewear. The inherent risk at a distance to the retina, cornea and lens is low, and this is attributable to beam attenuation consequent to the high beam divergence angle. For a professional prescribed device, it would be the best practice to supply better quality eyewear of defined characteristics as defined by the IEC. However, this may add to the cost to the consumer and is not at present a mandatory requirement.

**Future Requirements:** On consideration of the cost and logistical issues associated with the frequent re-treatment of some pathologies, self-administered LED devices may prove to be an important adjunct to clinical care. In order to achieve this, however, there will, by necessity, be the need to standardise dosimetry and accurate dose delivery. However, by design modifications and adequate supporting educational materials, this is not an overwhelming difficulty. As the evidence base develops, there may be a substantial increase in the uptake of home use LED devices. Although the risks associated with blue wavelengths are regarded as small, it can be reasonably expected that this may warrant a change in guidance in support of optical protection as a standard requirement.

The potential of this type of appliance as an adjunct via clinician-directed home care is notably exciting. In principle, this type of device may prove its worth and, given the relatively low cost to the consumer, may be adopted widely. However, given the lack of specific supporting evidence to promote this type of device, and the possibility of falling short with respect to the regulatory requirements of the EU and UK, it is our opinion that it is premature to offer professional endorsement, notwithstanding the apparent enthusiasm of consumers.

## 5. Conclusions

There are many possible future applications of home-based LED phototherapy devices to promote beneficial healing, relieve pain and inflammation, to permit home treatments for recurrent herpetic infections, as well as to enable novel usage for clinician-prescribed therapies. As promoted to the public at present, the LED devices available and included in this study fall far short of the need for the same. In respect to the torch type LED devices, the manufacturers provide very basic instructions to consumers. In the absence of evidence of compliance to the required regional regulatory standards for medical devices, to prescribe one of these devices may be a potential ‘trip-wire’ to a regulatory challenge with an associated medico-legal risk to a practitioner.

Although we view these devices as being of considerable potential benefit in dentistry, within the limits of our study, we can only offer an informed opinion. In view of the cost and logistical issues associated with the frequent re-treatment of some pathologies, self-administered LED devices may prove to be an important adjunct to clinical care. LED self-administered devices appear to offer great potential as an added tool to the clinical armamentarium. However, although the devices currently available may be amenable for these purposes, this must be subject to suitable scientific evaluation in order to establish a standardised evidence-based approach.

## Figures and Tables

**Table 1:** Consumer ratings, manual evaluation, proposed applications, beam divergence and critical feedback.

Device	Ratings 1–5* (%)	Av.rating	Cohort Size	Disclaimer?	Manual 0–5	Applications	Beam Divergence	Negative Feedback	Comments
1. Shenglaite (Shenzhen) (2.5 cm diameter)	5* 56 4* 26 3* 6 2* 4 1* 8	4.2	102	Y	2	Pain Myalgia TMD	74°	Poor instructions Equipment fault No benefit	Advice to use: apply surface-2.5 cm Video and printed images: hold at a distance
2. Yzoe Shiwang (Shenzhen) (2 cm diameter)	5* 38 4* 25 3* 13 2* 8 1* 15	3.7	402	N	1	Pain Myalgia TMD	74°	Poor instructions Equipment fault No benefit Excess heat	No advice on optical safety One report of smoke when charging
3. Truslite Joytisme (Shenzhen) (1 cm diameter)	5* 45 4* 15 3* 18 2* 6 1* 16	3.7	183	Y	3	Herpes Ulcers Skin (acne) Laryngitis	28°	No benefit Equipment fault	Many positive reports: rapid healing and pain mitigation with herpetic eruptions Device is limited to small areas: dubious benefit in laryngitis
4. Homesta (Shenzhen) (3 cm diameter)	5* 53 4* 12 3* 13 2* 2 1* 10	4.2	392	Y (USA) N (EU)	4	Pain Herpes Ulcers Tendonitis Arthritis Acne	74°	No benefit Equipment fault	Good access to consumer advice. However, rec.technique at variance to optimum parameters Kit includes tripod
5. Kinreen (Shenzhen) (3 cm diameter)	5* 70 4* 10 3* 6 2* 4 1* 10	4.3	272	Y (USA) N (EU)	3	Pain Herpes Ulcers Tendonitis Arthritis Acne	74°	No benefit Equipment fault	Many positive reports. Instruction manual basic

**Table 2:** Characterisation of devices included. All power readings recorded by measurement using PM160 Thor Labs optical power meter.

Device	λ	mW	mW/cm^2^	Time s.	Joules	Light Guide?	Glasses?	Gated Mode	CE/FDA?
1. Shenglaite (Shenzhen) (2.5 cm diameter)	460 630 660 850 900	All: 900	183	300	All: 270 Gated: 135	N	Y	10 Hz 50% Duty cycle 450 mW	CE: (electrical only) FDA: 510k (ILY)
2. Yzoe Shiwang (Shenzhen) (2 cm diameter)	630 660 850	450	144	300	43	N	N	N	CE: None FDA: None
3. Truslite Joytisme (Shenzhen) (1 cm diameter)	660 850	250/500	312/624	60	15/30	N	N	N	CE: (electrical only) FDA: None
4. Homesta (Shenzhen) (3 cm diameter)	460 630 660 850 900	All: 900 Red: 473 NIR: 335 Blue: 315 Gated: 450	All: 127.5 Red: 67 NIR: 47.5 Blue: 41 Gated:	300	All: 270 Red: 142 NIR: 100.5 Blue: 94.5 Gated: 135	All: 60 mW Red: 30 mW NIR: 10 Mw Blue: 10 mW	Y	10 Hz 50% Duty cycle 450 mW	CE: (electrical only) FDA: 510k (ILY)
5. Kinreen (Shenzhen) (3 cm diameter)	460 630 660 850 940	All: 750 Red+NIR: 511 Blue:242 Gated:450	All: 99 Red+NIR: 72 Blue: 34	180	All: 135 Red+NIR: 92 Blue: 43.6 Gated: 67.5	All: 50 mW Red+NIR: 35 mW Blue: 20 mW	Y	10 Hz 50% Duty cycle 375 mW	CE: (electrical only) FDA: 510k (ILY)

**Table 3:** The significance of χ^2^ contributions for contingency table cells containing Likert scores for the analysis model containing all separate studies recorded, encompassing four, two, two and two studies conducted on the Yzoe, Truslite, Homesta and Kinreen products. Statistically significant cells are detected using Fisher’s exact test (*p* < 0.05) and are displayed in red.

	1*	2*	3*	4*	5*
Shenglaite-1	<	<	**<**	>	>
Yzoe Study I	<	<	<	<	>
Yzoe Study II	>	**>**	**>**	>	**<**
Yzoe Study III	>	**>**	**<**	>	<
Yzoe Study IV	>	>	>	>	**<**
Truslite Study I	**>**	>	**<**	<	<
Truslite Study II	**<**	>	**>**	**<**	>
Homesta Study I	**<**	**<**	>	**>**	>
Homesta Study II	<	>	<	**<**	**>**
Kinreen Study I	<	<	<	<	>
Kinreen Study II	**<**	**<**	>	**>**	>

**Table 4:** The significance of χ^2^ contributions for contingency table cells containing Likert scores for the analysis model containing all summated results obtained for the Yzoe, Truslite, Homesta and Kinreen products. Statistically significant cells are detected using Fisher’s exact test *(p* < 0.05) and are displayed in red.

	1*	2*	3*	4*	5*
Shenglaite (1)	<	<	**<**	>	>
Yzoe (2)	**>**	**>**	>	>	**<**
TrusLite (3)	**>**	>	>	**<**	<
Homesta (4)	**<**	**<**	<	<	**>**
Kinreen (5)	**<**	**<**	>	>	>

## Data Availability

The original contributions presented in this study are included in this article. Further inquiries can be directed to the corresponding author.
